# CT-Based Quantification of Prostate Volume Change After LHRH-Agonist Androgen Deprivation: A Prospective, Three-Reader Study for Radiotherapy Planning

**DOI:** 10.3390/life16010029

**Published:** 2025-12-25

**Authors:** Nicolás Feltes Benítez, Manuel Galdeano-Rubio, Jesus Muñoz-Rodriguez, Arturo Domínguez, Josep Maria Solé i Monné, Meritxell Pérez Márquez, Sergio Caballero del Pozo, Inma Díaz-Álvarez, Felipe Couñago, Saturio Paredes-Rubio

**Affiliations:** 1 Department of Radiation Oncology, Hospital Universitario de Terrassa (CST), 08221 Terrassa, Spain; mgaldeano@cst.cat (M.G.-R.); jmsole@cst.cat (J.M.S.i.M.); scaballero@cst.cat (S.C.d.P.); idiaz@cst.cat (I.D.-Á.); sparedes@cst.cat (S.P.-R.); 2 Faculty of Medicine and Health Sciences, Universitat Internacional de Catalunya (UIC Barcelona), Campus Sant Cugat, 08195 Sant Cugat del Vallès, Spain; 3 Department of Urology, Corporació Sanitària Parc Taulí, Institut d’Investigació i Innovació Parc Taulí (I3PT-CERCA), Universitat Autònoma de Barcelona, 08208 Sabadell, Spain; jmunoz@tauli.cat (J.M.-R.); adominguez@tauli.cat (A.D.); 4 Department of Urology, Hospital Universitario de Terrassa (CST), 08221 Terrassa, Spain; mperez@cst.cat; 5 Department of Medicine, Faculty of Medicine, Health and Sports, Universidad Europea de Madrid, 28108 Madrid, Spain; fcounago@gmail.com; 6 Department of Radiation Oncology, Genesis Care Spain, Hospital Universitario San Francisco de Asís, 28002 Madrid, Spain; 7 Department of Radiation Oncology, Genesis Care Spain, Hospital Universitario La Milagrosa, 28010 Madrid, Spain

**Keywords:** prostate cancer, LHRHa, ADT, leuprolide, CT, radiotherapy planning, cytoreduction, organs at risk, PSA, testosterone

## Abstract

Introduction: ADT is routinely combined with radiotherapy (RT) for intermediate- and high-risk prostate cancer. While prostate shrinkage may facilitate planning, prospective CT-based, patient-level estimates over short, workflow-relevant intervals are scarce. Methods: We conducted a prospective study of 47 patients starting luteinizing hormone-releasing hormone agonist (LHRHa) therapy (leuprolide, 6-month depot). Prostate volumes were independently contoured by three blinded radiation oncologists on paired CT scans at baseline and ~8 weeks post-injection. The primary outcomes were the mean relative volume change and the proportion achieving a clinically relevant reduction (≥15%). PSA and testosterone were recorded at both time points; correlations and exploratory univariable logistic regression for ≥15% reduction were performed at the patient level. Results: Mean relative volume reduction ranged from −18.5% to −21.3% across observers; ≥60% of patients met the ≥15% threshold (RT-A 61.7%, RT-B 66.0%, RT-C 74.5%). PSA and testosterone decreased substantially (e.g., median PSA from 9.64 to 1.84 nmol/L) and were moderately correlated (Spearman ρ = 0.43, *p* = 0.002; Pearson r = 0.51, *p* < 0.001). No baseline clinical, histologic, or biochemical variables reached statistical significance for predicting ≥15% volume reduction; % PSA change showed a non-significant trend (OR 1.03; 95% CI 1.00–1.07; *p* = 0.076). Conclusions: Short-course LHRHa induced consistent CT-measured cytoreduction, with more than half of cases achieving ≥15% shrinkage within 8 weeks. Prostate downsizing was reproducible across readers and accompanied by marked PSA and testosterone declines, although biochemical responses did not predict volumetric change. These findings support incorporating a short neoadjuvant “window” before RT simulation and highlight the need for larger studies to refine predictors and compare agonist vs. antagonist trajectories.

## 1. Introduction

Prostate cancer is the most frequently diagnosed malignancy (excluding non-melanomatous skin cancer) in men worldwide; in Spain, 32,967 new cases were recorded in 2022 [1]. For men with unfavorable-intermediate to very-high-risk localized disease, combining androgen deprivation therapy (ADT) with radiotherapy (RT) is endorsed across major clinical guidelines [2,3,4]. Although adding ADT to RT consistently improves survival in these groups, the optimal sequencing of ADT relative to RT (neoadjuvant vs. concurrent/adjuvant) remains debated [5,6].

Beyond systemic control, ADT can shrink the prostate, yet the magnitude, timing, and inter-patient variability of this anatomical change are not well defined in a way that is directly actionable for RT planning [7]. Prior reports mixed modalities and schedules—e.g., ultrasound pre-ADT then CT post-ADT, or mpMRI pre-ADT then CT for planning—so pre/post volume was rarely measured with the same method, limiting validity for simulation workflows [8,9]. In routine practice, by contrast, CT is the universal, workflow-defining platform within RT departments: widely available, standardized across centers, and ultimately the imaging on which target and OAR contours are finalized.

Contemporary randomized evidence further suggests that when short-course ADT is combined with prostate-only RT, concurrent/adjuvant sequencing yields superior long-term oncologic outcomes compared with a neoadjuvant-first approach of equal duration (e.g., improved progression- or metastasis-free survival without excess grade ≥3 GI/GU toxicity) [5,6]. This evidence base creates a practical opportunity for a brief “mini-neoadjuvant” window—i.e., a short, time-limited interval to capture CT-quantified downsizing that facilitates dosimetry and OAR sparing—while preserving the oncologically favored concurrent/adjuvant backbone thereafter. However, standardized CT-based data specifying how much and how fast prostates shrink over short, workflow-relevant intervals remain scarce.

Against this background, we conducted a prospective evaluation of LHRH-agonist ADT (Leuprolide), using paired CT at ~8 weeks with independent delineations by three observers to (i) quantify prostate volume change at the patient level, (ii) estimate the proportion achieving a pre-specified clinically relevant reduction (≥15%), and (iii) explore who experiences shrinkage, when shrinkage occurs, and how much shrinkage occurs by relating volumetric change to concurrent PSA/testosterone dynamics and baseline clinical factors. By anchoring the analysis in CT—the modality that ultimately guides RT delivery—this study aims to provide immediately translatable evidence to refine simulation timing and personalize treatment planning.

## 2. Materials and Methods

### 2.1. Study Design

A prospective observational study was conducted including a cohort of 47 patients with localized PC, all evaluated by multidisciplinary oncologic committees. For those cases in which a combined treatment strategy with ADT and RT was recommended, patients were invited to participate during their first visit to the Radiation Oncology Department.

Patient recruitment took place between October 2023 and October 2024. All patients met predefined inclusion and exclusion criteria (available in the Supplementary Materials). The study protocol was reviewed and approved by the Scientific Committee of the Hospital Universitario de Terrassa and received ethics approval from the Institutional Review Board (CEIm) under code 01-23-100-099. The study is also registered at ClinicalTrials.gov under ID NCT06184464. All participants signed informed consent in accordance with ethical and regulatory standards.

### 2.2. Procedure

Hormonal treatment consisted exclusively of leuprolide acetate in atrigel formulation (Eligard^®^, 6-month depot), with no prior administration of oral antiandrogens (e.g., bicalutamide).

Each patient underwent two computed tomography (CT) scans:

The first CT scan was performed on Day 1 of the study, during a single visit to the Radiation Oncology Department. On that day, patients underwent blood sampling for PSA and testosterone, followed by the simulation CT scan, and finally Eligard^®^ was administered by the department’s nursing staff. This baseline scan constituted the only study-specific procedure outside routine clinical practice.

The second CT scan was performed two months after Eligard^®^ injection and corresponds to the standard scan used for target volume delineation prior to RT planning.

Both baseline and post-ADT scans were performed using CT with 3-mm slices, in line with international prostate RT planning guidelines (RTOG). Although mpMRI is routinely used for initial diagnostic and staging purposes, post-ADT mpMRI is not part of standard simulation workflows in our institution and is therefore not systematically acquired. To ensure consistency and direct applicability to real-world RT planning workflows, volumetric assessment at both time points was based exclusively on CT.

The prostate gland was independently contoured by three radiation oncologists, using the Xio^®^ and Focal-Monaco^®^ (Elekta) treatment planning systems with individualized login access. Each physician performed contouring without access to the contours created by the other observers, thus ensuring inter-observer blinding and independence. All three observers were board-certified radiation oncologists with more than five years of experience in genitourinary oncology; the senior observer had over a decade of experience in prostate imaging and contouring.

Each observer completed the delineation within 10 days after each scan, using available clinical information and following RTOG guidelines for contouring the prostate and organs at risk (rectum and bladder).

**Figure 1:**
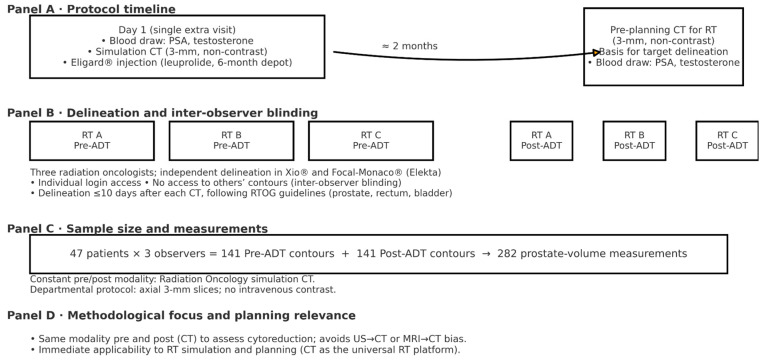
Study schema and workflow.

Given the inclusion of 47 patients and the participation of three observers per patient, a total of 141 pre-ADT and 141 post-ADT prostate contours were obtained, yielding 282 volumetric measurements. The study workflow is summarized in Figure 1.

### 2.3. Collected Variables

The following clinical and pathological variables were collected prior to radiotherapy:

Medical history (including hypertension, diabetes, dyslipidemia, cardiovascular disease, and combinations).

PSA level at time of diagnostic biopsy.

Date of biopsy.

Histologic data by lobe: Gleason score, number of affected cores, and total number of cores (right and left lobes).

Prostate volume (in cubic centimeters) as estimated by transrectal ultrasound.

Prostate volume (in cubic centimeters) as estimated by multiparametric MRI.

Presence of perineural invasion.

Radiologic classification from multiparametric MRI.

Risk group classification (low, intermediate, high).

In addition, the following study-specific variables were recorded:

- Date of first visit.
- Date of pre-ADT (baseline) simulation CT scan.
- Date of post-ADT simulation CT scan.
- PSA and testosterone levels prior to Eligard^®^ injection. Serum testosterone was measured using the institutional laboratory assay and reported in nmol/L (reference range 6.68–25.7 nmol/L).
- PSA and testosterone levels prior to radiotherapy planning (2 months post-ADT).
- Prostate volume according to each observer on both pre- and post-ADT CT scans.
- Acute genitourinary and gastrointestinal toxicities, classified according to CTCAE v5.0.

### 2.4. Follow-Up

After treatment completion, patients will continue routine follow-up every 6 months through either Urology or Radiation Oncology outpatient clinics, up to 10 years from the time of diagnosis, in accordance with institutional protocols.

Follow-up evaluations will include monitoring for biochemical recurrence, as well as early and late toxicities potentially related to RT or ADT. The time to testosterone recovery will also be tracked as a secondary outcome.

### 2.5. Sample Size Justification

At the time of study design, no prior references were available regarding the expected variability in percent prostate-volume change after ADT, nor was a validated threshold of clinically meaningful reduction. We pre-specified that a ≥15% reduction would be considered clinically relevant, as prior neoadjuvant ADT series consistently report prostate shrinkage in this magnitude range, and because such a threshold provides a pragmatic and interpretable benchmark for differentiating minimal changes from clearly meaningful cytoreduction in the context of radiotherapy planning.

Percent change was defined as:

%Δ = post-ADT volume-baseline volume × 100 (negative values indicate reduction)

                               baseline volume     

Based on conservative assumptions (SD ≈ 40% at the patient level), a sample of 47 patients yields an estimated standard error of ≈ 5.8% and a 95% CI half-width of ≈ 11.4% for the mean % change, which we deemed acceptable for an exploratory estimation study. Multiple delineations per patient (total 282) increase precision for variance components and enable robust inter-observer agreement assessment (ICC) but are not counted as independent patients.

For context only, a traditional hypothesis-testing framework would require ~128 patients for 80% power to detect a mean change of −15% vs. 0% (two-sided α = 0.05), which informed future confirmatory planning.

### 2.6. Statistical Analysis

Analyses were performed at the patient level. Categorical variables were reported as absolute and relative frequencies (%), and continuous variables as mean ± SD or median (IQR), depending on distribution. Paired baseline vs. post-ADT comparisons used the paired *t*-test or, when normality was not supported (Shapiro–Wilk), the Wilcoxon signed-rank test. Categorical variables were compared using the chi-square test or Fisher’s exact test. Statistical significance was set at *p* < 0.05. Analyses were performed using IBM SPSS Statistics version 29.

## 3. Results

### 3.1. Baseline Characteristics

A total of 47 patients with localized prostate cancer were included. The median age at the time of the first visit was 76 years (IQR: 72–79; range: 55–86). The median PSA at diagnosis was 9.64 ng/mL. Baseline characteristics of the study population are summarized in Table 1

The prostate volume measured by multiparametric MRI had a median of 46.0 cc (IQR: 35.5–63.2), with values ranging between 20 and 113 cc.

Perineural invasion was reported in 34.0% of patients.

In terms of histological classification, the distribution according to the ISUP system was as follows: ISUP 1 (Gleason 3+3): 5 patients (10.6%); ISUP 2 (Gleason 3+4): 20 patients (42.6%); ISUP 3 (Gleason 4+3): 15 patients (31.9%); ISUP 4 (Gleason ≥ 4+4): 7 patients (14.9%).

With respect to clinical risk group stratification, 25 patients (53.2%) were classified as intermediate risk and 22 patients (46.8%) as high risk, based on guideline-concordant definitions.

### 3.2. Prostate Volume Before and After LHRHa

Before the initiation of LHRHa treatment, the mean prostate volume varied across observers, ranging from 42.6 cc (RT A) to 55.4 cc (RT C), reflecting expected inter-reader differences despite standardized contouring guidelines. After two months of treatment with Eligard^®^, all observers documented consistent cytoreduction. Mean relative volume reductions were 18.5% for RT A, 21.3% for RT B, and 19.4% for RT C, with all median reductions exceeding the predefined ≥15% threshold. Detailed pre- and post-ADT values for each observer are provided in Table 2.

### 3.3. Proportion of Patients with ≥15% Prostate Volume Reduction

The number of patients achieving a ≥15% reduction in prostate volume was analyzed for each of the three independent observers. This threshold was used to identify patients who demonstrated a clinically relevant anatomical response following ADT. The results are shown in Table 3.

According to RT A, 29 out of 47 patients (61.7%) achieved a reduction of ≥15%. RT B identified 31 patients (66.0%), and RT C identified 35 patients (74.5%) meeting or exceeding this threshold. The remaining patients did not reach the 15% reduction threshold and were classified as non-responders.

Importantly, the ≥15% threshold was applied independently to each observer’s paired contours. As expected, the sets of patients classified as responders showed substantial but not complete overlap across RT A, RT B, and RT C, reflecting normal inter-observer variability in baseline and post-ADT contouring.

### 3.4. Reduction in PSA and Testosterone Following Hormonal Therapy

PSA and testosterone values were analyzed both before the initiation of therapy with Eligard^®^ and two months after administration. The results are shown in Table 4, including means, medians, and reduction ranges.

The median baseline PSA was 9.64 ng/mL, with a mean of 14.91 ng/mL. After treatment, PSA decreased to a median of 1.84 ng/mL and a mean of 2.95 ng/mL. The mean percentage reduction in PSA was 76.1% ± 18.9, with a median of 79.1%.

**Figure 2:**
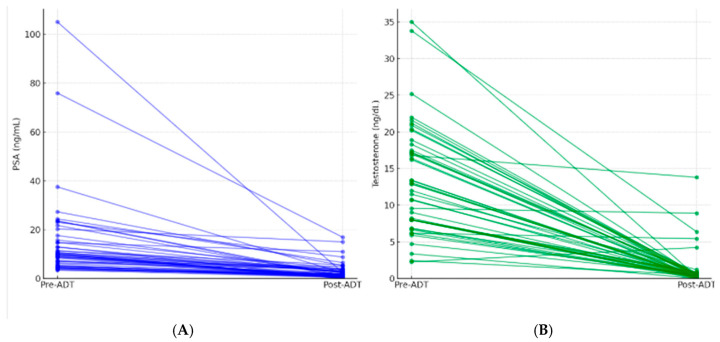
(**A**). Individual PSA response to short-course LHRH agonist (paired pre- vs. post-ADT). (**B**). Individual testosterone suppression after short-course LHRH agonist (paired pre- vs. post-ADT).

For testosterone, the mean pre-treatment level was 13.2 nmol/L (median: 12.0), while the post-treatment value dropped to a mean of 1.15 nmol/L (median: 0.39). The mean percentage reduction in testosterone was 86.6% ± 33.4%, with a median of 97.1%. At two months, 42 of 47 patients (89.4%) achieved biochemical castration, defined as testosterone <1.7 nmol/L. Summarized in Figure 2. 

Additionally, the proportion of patients reaching different thresholds of hormonal reduction was calculated. These results are summarized in Table 5.

### 3.5. Correlation Between PSA and Testosterone Reduction

A correlation analysis was performed to assess whether the percentage reduction in PSA levels was associated with the percentage reduction in testosterone following ADT. Both Spearman’s (non-parametric) and Pearson’s (parametric) correlation coefficients were calculated using data from all evaluable patients with complete hormonal data. These results are summarized in Table 6.

The analysis revealed a moderate and statistically significant positive correlation between the two variables. Specifically, patients who experienced greater reductions in testosterone levels tended to also exhibit more pronounced reductions in PSA.

These findings suggest a proportional relationship between the degree of androgen suppression and the decline in PSA levels in response to LHRHa therapy.

#### Univariable Logistic Regression Analysis

A univariable logistic regression analysis was conducted to explore potential predictors of achieving a ≥15% reduction in prostate volume following ADT. The independent variables included age, baseline PSA, percentage reduction in PSA, baseline testosterone, percentage reduction in testosterone, MRI-based prostate volume, percentage of positive biopsy cores, presence of perineural invasion (PNI), ISUP Gleason grade, and clinical risk group, no variable reached *p* < 0.05.

However, a trend toward association was observed for percentage reduction in PSA, which approached statistical significance (*p* = 0.076), suggesting a potential relationship to be explored in future analyses with larger cohorts. Given the marked biochemical suppression observed in this cohort, we also examined castration achievement (<1.7 nmol/L) as an exploratory predictor. Although it did not reach statistical significance, it represents an increasingly relevant biological parameter—particularly with the emergence of fast-acting oral GnRH antagonists—and warrants evaluation in larger datasets.

Complete results are summarized in Table 7.

## 4. Discussion

Our prospective evaluation focused on the magnitude and consistency of prostate volume change with LHRH-agonist ADT (leuprolide), assessed by paired CT at ~8 weeks and replicated across three independent readers. We observed a coherent cytoreductive signal—mean relative volume reductions of ~18–21% and ≥60% of patients reaching the pre-defined ≥15% threshold—accompanied by substantial PSA and testosterone declines and a moderate correlation between their percent changes, indicating aligned anatomical and biochemical effects.

Beyond documenting shrinkage, these findings are clinically meaningful for RT planning. Smaller prostate volumes can improve target coverage and reduce the likelihood that bladder and rectum are encompassed within high- or intermediate-dose regions, thereby supporting more favorable dose–volume metrics [7]. The choice of ≥15% as a clinically meaningful volumetric threshold warrants comment. This threshold was defined a priori as an anatomical endpoint, as dosimetric verification was not part of the predefined objectives of this exploratory study. Previous neoadjuvant ADT series have generally reported prostate shrinkage within the 15–30% range [8,9,10,11], supporting the use of a threshold within this interval for characterizing cytoreduction. In addition, for an exploratory estimation study, a ≥15% cut-off provides a pragmatic and clinically interpretable benchmark, allowing us to differentiate minor volumetric variations from reductions likely to influence radiotherapy planning and organ-at-risk dosimetry. Although arbitrary to some extent, this threshold aligns with published evidence and offers a reasonable balance between clinical relevance and methodological feasibility.

Consistent with this rationale, prior work has linked neoadjuvant ADT to improved dosimetry and lower toxicity in IMRT settings [9,10]. Although the magnitude of cytoreduction observed here (~8 weeks) is lower than that reported with longer courses, the direction and consistency across observers indicate that even time-limited volume reduction can be leveraged to optimize planning and potentially mitigate adverse effects.

These results are also consistent with literature describing ADT-associated prostate shrinkage, with reported magnitudes comparable to those seen here. Langenhuijsen et al. documented substantial reductions over approximately three months with smaller additional changes thereafter, supporting the pragmatic use of time-limited courses when pre-radiotherapy cytoreduction is intended [11]. Studies addressing lower urinary tract symptoms (LUTS) further suggest that anatomical shrinkage can translate into symptomatic benefit, particularly in patients with moderate–severe baseline burden, albeit with domain-specific variability [12,13]. While we did not capture LUTS or quality-of-life outcomes, baseline symptom profiling remains relevant when tailoring ADT duration and expectations.

In routine practice, treatment planning is CT-based, and many centers cannot systematically acquire mpMRI at 2–3 months. While mpMRI remains central for tumor characterization, its functional contrasts may diminish after ADT, potentially reducing lesion conspicuity [12]. Our CT-centric approach reflects real-world workflows and underscores a practical point: biochemical response is not a surrogate for volumetric response. In this cohort, PSA and testosterone fell markedly and correlated, yet neither independently predicted achieving the ≥15% cytoreduction threshold. This decoupling has implications for scheduling and for how clinicians infer anatomical change from laboratory kinetics; volumetric assessment should not be assumed based solely on PSA suppression.

Regarding predictors of cytoreduction, previous work has highlighted baseline prostate volume as a key determinant [14]. In our exploratory univariable analysis—including clinical, histologic, and biochemical variables—no factor reached statistical significance for predicting a ≥15% volume reduction; the trend for percent PSA change (*p* = 0.076) is hypothesis-generating and merits testing in larger datasets and multivariable models. Multivariable modeling was not performed because the sample size did not allow stable multi-parameter estimation without risking overfitting; therefore, univariable analyses were used in keeping with the exploratory aims of the study. In this context MRI-derived prostate volume was used as the baseline covariate because it represented a standardized, observer-independent measurement available for all patients, whereas TRUS volumes were obtained under heterogeneous operator- and device-dependent conditions and were therefore not included. The absence of association between baseline volume and ≥15% reduction in our study likely reflects the modest sample size, the relatively narrow range of baseline prostate volumes, and the short 8-week interval. Early ADT response is predominantly driven by epithelial atrophy, while baseline gland size becomes a stronger predictor at later time points reported in prior studies. These methodological and biological factors may explain why our findings differ from some earlier reports. Prostate gland volume is anatomically independent of bladder and rectal filling; although physiological variations in pelvic filling are expected, the high inter-observer consistency in relative volume reduction supports that the observed cytoreduction reflects true glandular change rather than preparation variability.

The strengths of this study include its prospective design; paired imaging with standardized contouring; three independent, blinded readers supporting reproducibility; and homogeneous pharmacologic exposure with depot leuprolide.

Crucially, these planning-centric findings can be integrated with the survival-focused evidence base: a short neoadjuvant window to capture CT-verified downsizing for simulation, immediately followed by concurrent/adjuvant ADT in line with randomized/meta-analytic data [5,6]. This hybrid, workflow-aware strategy reconciles anatomical optimization with evidence-based systemic timing and may help standardize simulation schedules in everyday practice.

### 4.1. Limitations

LUTS and quality-of-life outcomes were not collected, restricting clinical interpretation beyond anatomical and biochemical responses. The moderate sample size limits power for predictor discovery and precludes robust multivariable modeling. Inter-observer variability—although expected and explicitly reported—underscores the need for further standardization and for advanced segmentation tools. In our cohort, bicalutamide was not administered because none of the patients had metastatic disease, risk of spinal cord compression, or clinically significant obstructive or voiding symptoms—situations in which flare prevention is indicated. This approach also avoided introducing additional pharmacologic cofactors, ensuring a more homogeneous assessment of leuprolide-induced biochemical and volumetric changes.

This manuscript presents the pre-specified ≈8-week analysis in the initial cohort. The planned 3-month CT comparison pertains to a subsequent phase of the project involving a new cohort currently under recruitment.

### 4.2. Future Directions

Building on these data, we are expanding the cohort to refine predictors of cytoreduction and to evaluate whether newer LHRH antagonists, which often induce a more rapid PSA decline, display distinct early volumetric trajectories. Prospective designs that integrate standardized functional outcomes (e.g., LUTS scales) and leverage imaging- and AI-assisted segmentation should enhance volumetric assessment and response prediction, ultimately enabling more precise, patient-tailored radiotherapy planning.

## Figures and Tables

**Table 1:** Baseline characteristics of the study population (n = 47).

Variable	Value
Age (years)	76.0 (IQR: 72.0–79.0)
PSA at diagnosis (ng/mL)	9.64 (IQR: 5.98–15.40)
Prostate volume by MRI (cc)	46.0 (IQR: 35.5–63.2)
Perineural invasion	34.0%
ISUP 1 (Gleason 3+3)	5/47 (10.6%)
ISUP 2 (Gleason 3+4)	20/47 (42.6%)
ISUP 3 (Gleason 4+3)	15/47 (31.9%)
ISUP 4 (Gleason ≥ 4+4)	7/47 (14.9%)
Risk group: Intermediate	25/47 (53.2%)
Risk group: High	22/47 (46.8%)

Footnotes: Values are median (IQR) or n/N (%), unless otherwise specified.

**Table 2:** Prostate volume before and after ADT by observer (n = 47).

	RT A	RT B	RT C
Prostate volume pre-ADT (mean ± SD, cc)	42.6 ± 17.4	50.4 ± 22.2	55.4 ± 24.3
Prostate volume post-ADT (mean ± SD, cc)	35.2 ± 15.7	39.7 ± 18.4	44.7 ± 20.4
Absolute volume reduction (cc)	7.4	10.7	10.6
Mean relative reduction (%)	18.5 ± 11.2	21.3 ± 10.4	19.4 ± 9.3
Median relative reduction (%)	16.1	20.6	18.8

Footnotes: Relative change defined as (post − baseline)/baseline × 100; negative values indicate reduction (negative values indicate reduction; “relative change” defined as (post − baseline)/baseline × 100).

**Table 3:** Proportion of patients achieving ≥15% prostate volume reduction according to observer (n = 47).

Observer	Patients with ≥15% Reduction	Patients with <15% Reduction	% with ≥15% Reduction
RT A	29	18	61.7%
RT B	31	16	66.0%
RT C	35	12	74.5%

**Table 4:** PSA and testosterone before and after ADT (n = 47).

Parameter	Mean ± SD	Median	Min–Max
PSA pre-ADT (ng/mL)	14.91 ± 17.96	9.64	3.46–105.00
PSA post-ADT (ng/mL)	2.95 ± 3.55	1.84	0.02–16.90
% PSA reduction	76.1 ± 18.9%	79.1%	25.2%–99.8%
Testosterone pre-ADT (nmol/L)	13.21 ± 7.39	12.00	2.26–35.00
Testosterone post-ADT (nmol/L)	1.15 ± 2.57	0.39	0.02–13.80
% Testosterone reduction	86.6 ± 33.4%	97.1%	−87.2%–99.8%

Footnotes: Units per laboratory reporting; percent change as above.

**Table 5:** Proportion of patients by prespecified hormonal reduction thresholds.

Hormonal Reduction Category	% of Patients
PSA reduction ≥75%	59.6%
PSA reduction ≥90%	23.4%
Testosterone reduction ≥85%	87.2%
Testosterone reduction ≥90%	78.7%

**Table 6:** Correlation between % reduction in PSA and testosterone (n = 47).

Correlation Test	Correlation Coefficient (r)	*p*-Value
Spearman	0.43	0.002
Pearson	0.51	<0.001

**Table 7:** Univariable logistic regression for predictors of ≥15% prostate volume reduction.

Variable	Odds Ratio (OR)	95% CI	*p*-Value
Age	0.97	0.91–1.04	0.289
PSA at diagnosis	0.99	0.96–1.03	0.352
% PSA reduction	1.03	1.00–1.07	0.076
Baseline testosterone	1.01	0.91–1.13	0.815
% Testosterone reduction	1.01	0.99–1.03	0.183
Prostate volume by MRI	0.99	0.96–1.03	0.617
Perineural invasion (PNI)	1.73	0.45–6.72	0.424
% positive biopsy cores	0.99	0.95–1.03	0.576
Highest ISUP Gleason grade	0.88	0.53–1.47	0.625
Clinical risk group	1.11	0.57–2.15	0.759

## Data Availability

Data supporting the findings of this study are available from the corresponding author on reasonable request, subject to institutional approvals and data-protection regulations.

## Supplementary Materials

The following supporting information can be downloaded at: https://www.mdpi.com/article/10.3390/life16010029/s1. Refs. [15,16,17,18,19,20,21,22,23] has been cited in Supplementary Materials.

## Abbreviations

ADT	Androgen deprivation therapy
CT	Computed tomography
GI	Gastrointestinal
GU	Genitourinary
IMRT	Intensity-modulated radiotherapy
LHRH	Luteinizing hormone-releasing hormone
LHRHa	Luteinizing hormone-releasing hormone agonist
LLOQ	Lower limit of quantification
LUTS	Lower urinary tract symptoms
MFS	Metastasis-free survival
mpMRI	Multiparametric magnetic resonance imaging
OAR	Organ at risk
PC	Prostate Cancer
PSA	Prostate-specific antigen
RT	Radiotherapy
