# Supplementary material for: CT-Based Quantification of Prostate Volume Change After LHRH-Agonist Androgen Deprivation: A Prospective, Three-Reader Study for Radiotherapy Planning
Source: Life (Basel). 2025 Dec 25;16(1):29. doi: 10.3390/life16010029 (PMC12843479; doi:10.3390/life16010029)

Cito reducción prostática tras la administración de Eligard (Acetato de Leuprorelina) en el cáncer de próstata localizado previo al Tratamiento con Radioterapia. Estudio Piloto

1. INTRODUCCIÓN

El cáncer de próstata (CP) es el tumor más frecuente en hombres en el mundo, también en nuestro país con una incidencia de 34413 casos en el año 2020 (70.6 por cada 100000 habitantes) [1].
La elevación en sangre del PSA (Antígeno Prostático Sérico) y/o una alteración en el tacto rectal nos obligan a realizar una biopsia prostática que es la forma de diagnosticar el CP, procedimiento donde se extrae una pequeña muestra de tejido prostático para analizar en un microscopio y clasificar las células cancerosas según la escala de Gleason (grado de malignidad).
Una vez diagnosticado, se procede a realizar los llamados estudios de extensión (TAC toraco-abdominal, Gammagrafía Ósea, PET COLINA, etc.) que permiten descartar la diseminación a otros órganos (metástasis), de las células tumorales originadas en la próstata.
La gran mayoría de los CP se diagnostican en estadios localizados (sin enfermedad a distancia) lo que permite plantear tratamientos locales con intención curativa, entre los cuales tenemos la cirugía (Prostatectomía) y el tratamiento con radiaciones (Radioterapia Prostática).
El bloqueo androgénico hormonal (BAH) en combinación con Radioterapia (RT) mejora los resultados oncológicos del CP localizado [2-3] pero además la utilización de BAH previo a la RT genera una cito reducción de la glándula prostática tras su administración [4-5].
Esta cito reducción de volumen de la próstata permite disminuir el volumen a irradiar y por ende con esto una menor toxicidad sobre tejidos y órganos sanos circundantes (vejiga y recto principalmente)
Esta aparente cito reducción se produciría a los dos meses de iniciado el BAH momento en el cual realizamos habitualmente el TAC de simulación del tratamiento radioterápico, pero no existen estudios que certifiquen ni cuantifiquen esta reducción ni sus consecuentes beneficios en el tratamiento del CP.
Actualmente es muy difícil de evaluar y comparar el tamaño de la próstata pre y post BAH, ya que la primera medición se obtiene en la ecografía y/o la resonancia realizada al momento diagnóstico y la segunda medición (post BAH) solo se realiza al momento de la planificación por parte del Oncólogo radioterápico en un TAC.
Por lo cual para una comparativa de tamaño nos encontramos frente a dos problemas, el primero es lo inadecuado que resulta de comparar tamaños prostáticos entre pruebas distintas como son la ecografía prostática, la resonancia y el TAC utilizado en la planificación.
Y segundo las posibles discordancias en la delimitación de la glándula prostática a partir de las posibles variabilidades en la delimitación por parte de los diferentes Oncólogos Radioterápicos que formaran parte del estudio [7]

2 OBJETIVOS

El objetivo **general** del estudio es: Valorar la reducción del tamaño prostático en TAC tras la administración del BAH, en pacientes con CP localizado que vayan a recibir tratamiento con radioterapia
Como **objetivos secundarios**: Confirmar posibles beneficios clínicos-dosimétricos (entendiendo que una reducción en el tamaño prostático implicara una menor inclusión en el volumen de tratamiento del recto y/o vejiga y consecuentemente una reducción en las toxicidades por esto generadas.
Evaluar la concordancia entre Oncólogos Radioterápicos en la determinación del tamaño prostático en el TAC de planificación del tratamiento radioterápico.

3 HIPOTESIS

3a. Este estudio permitirá confirmar una disminución en el tamaño prostático (en cc) a los dos meses de la administración del tratamiento Hormonal.
3b. Este estudio permitirá cuantificar la reducción conseguida tras el BAH y los posibles beneficios dosimétricos y clínicos (reducción de PTV dentro del recto y vejiga).

4 MATERIALES Y MÉTODOS

4a CRITERIOS DE INCLUSIÓN

-Confirmación histológica de adenocarcinoma de próstata por biopsia.
-CP localizado candidatos a tratamiento con BAH y radioterapia valorados en comités multidisciplinar.

-Formulario de consentimiento informado firmado.

4b CRITERIOS DE EXCLUSION
- Tamaño prostático por pruebas diagnostica de menos de 20 cc.
- Ganglios linfáticos positivos o enfermedad metastásica de cáncer de próstata en estudios de imagen.
-Radioterapia pélvica previa.

4c BASE DE DATOS A RELLENAR (40 pacientes)
General

- Variables clínico-patológicas previas al tratamiento de radioterapia externa:

Edad, Ant médicos, grado de malignidad microscópico mediante escala de Gleason obtenido en la biopsia diagnóstica.
Estadio clínico mediante Tacto rectal y/o Resonancia magnética.

- Variables del tratamiento de radioterapia externa aplicado:

Dosis total administrada en Gray.
Duración total en días de cada uno de los esquemas de tratamiento
Campo de irradiación: sólo próstata, próstata y vesículas seminales o agregando irradiación pélvica

Especificas del estudio

- Fecha primera visita
- Fecha TAC simulación pre-BAH
- Fecha aplicación BAH
- PSA y Testosterona al diagnóstico (pre-BAH)
- Tamaño prostático en centímetros cúbicos (cc)obtenidos de la resonancia o ecografía al diagnóstico de la delimitación por parte de los Oncólogos Radioterápicos (pre-BAH)
- Tamaño prostático en centímetros cúbicos (cc) obtenidos en la delimitación por los Oncólogos Radioterápicos (post-BAH)
- PSA y Testosterona previo a tratamiento radioterápico (post-BAH)
- Tamaño prostático en centímetros cúbicos (cc) obtenidos en la delimitación por los Oncólogos Radioterápicos (post-BAH)
- *Toxicidades agudas Vesicales y rectales recogidas según CTCAE 5.0[8]

Inclusión de 40 pacientes diagnosticados de cáncer de próstata localizado provenientes de los comités oncológicos que cubre el servicio de Oncología Radioterápica del Hospital de Terrassa y siempre que se haya decidido un tratamiento con BAH (Eligard semestral) y radioterapia con intención curativa sobre los mismos según práctica clínica habitual
Los comités Uro-oncológicos son: Comité Uro-Oncológico del Hospital de Terrassa, Comité Uro-Oncológico Hospital Mutua de Terrassa, Comité Uro-Oncológico Hospital Parc Taulí y Comité Uro-Oncológico Hospital de San Juan de Dios-Fundación Althaia.
Ya en la primera visita al Servicio de Oncología Radioterápica se invitará al paciente que haya cumplido los criterios de inclusión a participar del estudio y a la firma del Consentimiento informado aprobado por el CEIM
Se procederá a la realización del TAC de simulación previo al inicio del BAH y un segundo TAC (en el que habitualmente delimitamos los volúmenes a irradiar y los órganos de riesgo) a los dos meses del BAH.
La dosis efectiva recibida por un paciente en un estudio de CT estándar de pelvis es de aproximadamente 10mSv [9-10].Si comparamos estos valores con la dosis total de un tratamiento de radioterapia estándar de próstata (entre 60Gy y 70Gy), esto representa alrededor de un 0.9-1%.

En cada uno de esos TAC, la delimitación de la glándula prostática será realizada por los 4 Oncólogos Radioterápicos expertos en la patología prostática y responsables de los comités previamente nombrados con el objetivo de estudiar la concordancia en la delimitación en la glándula prostática entre los mismos.

La delimitación de cada paciente deberá realizarse con un máximo de 10 días desde la realización de cada uno de los TAC (pre BAH y post BAH) utilizando para la misma los datos de la historia clínica de los pacientes y guiados en la delimitación por consensos validados para la misma [(6)]
Se utilizarán herramientas propias del sistema de planificación Xio que permiten la absoluta individualización de cada paciente : manteniendo oculta la planificaciones realizadas sobre cada paciente por el resto de los Oncólogos Radioterápicos en favor del estudio de variaciones inter observador si las hubiese.
Los médicos responsables de cada comité Uro-Oncológico son: Dr. Feltes, Nicolas (Hospital de Terrassa y Hospital Parc Tauli), Dr. Galdeano, Manuel (Hospital de SJdD-Fundación Althaia), Dr. Lozano Joan (Hospital Mutua de Terrassa) y Dr. Paredes, Saturio (Hospital Parc Tauli).
La aplicación de BAH (Eligard) será llevada a cabo por enfermera de cada centro.
El cálculo dosimétrico será responsabilidad del Equipo de Dosimetría y Física médica.

Análisis estadístico:

Se presentarán las frecuencias absolutas y relativas para el análisis descriptivo de las variables cualitativas, la media con la desviación estándar o mediana y rango intercuartílico para la descripción de las variables cuantitativas. Para la comparación de variables cualitativas se utilizará la chi-cuadrado o la prueba exacta de Fisher. Para la comparación de medias se utilizará la t de Student y en caso de no cumplir condiciones de aplicación se utilizará la correspondiente prueba no paramétrica. Para el análisis de las concordancias Inter observadores se utilizará el coeficiente de correlación intraclase. El nivel de significación estadístico considerado para el análisis será del 5%. Los datos se analizarán con el programa estadístico SPSS v29.

5. BIBLIOGRAFIA

1 Global Cancer Observatory. Estimated age-standardized incidence rates (World) in 2020, Spain, both sexes, all ages.

2. D'amico et al. Androgen Suppression and Radiation vs Radiation Alone for Prostate Cancer A Randomized Trial. JAMA, January 23, 2008.

3. Lawton et al. Duration of Androgen Deprivation in Locally Advanced Prostate Cancer: Long-Term Update o NRG Oncology RTOG 9202. Int J Radiation Oncol Biol Phys, Vol. 98, No. 2, pp. 296e303, 2017.

4. Jethwa KR et al., Predictors of prostate volume reduction following neoadjuvant cytoreductive androgen suppression. J Contemp Brachytherapy. 2016

5. Solhjem MC et al., Prostate volume before and after permanent prostate brachytherapy in patients receiving neoadjuvant androgen suppression. Cancer J. 2004

6. Salembier C, Villeirs G, De Bari B, Hoskin P, Pieters BR, Van Vulpen M, Khoo V, Henry A, Bossi A, De Meerleer G, Fonteyne V. ESTRO ACROP consensus guideline on CT- and MRI-based target volume delineation for primary radiation therapy of localized prostate cancer. Radiother Oncol. 2018 Apr;127(1):49-61. doi: 10.1016/j.radonc.2018.01.014. PMID: 29496279.
7. Métodos estadísticos de evaluación de la concordancia y la reproducibilidad de pruebas diagnósticas. September 2010Revista Colombiana de Obstetricia y Ginecología 61(3):247-255.DOI: 10.18597/rcog.271
8.CTCAE 5.0
https://ctep.cancer.gov/protocoldevelopment/electronic_applications/docs/ctcae_v5_quick_reference_5x7.pdf

9.Kan, Monica & Leung, Lucullus & Wong, Wicger & Lam, Nelson. (2008). Radiation Dose From Cone Beam Computed Tomography for Image-Guided Radiation Therapy. International journal of radiation oncology, biology, physics. 70. 272-9. 10.1016/j.ijrobp.2007.08.062.

10. Parham Alaei, Emiliano Spezi, Imaging dose from cone beam computed tomography in radiation therapy, Physica Medica, Volume 31, Issue 7, 2015, Pages 647-658, ISSN 1120-1797, https://doi.org/10.1016/j.ejmp.2015.06.003.

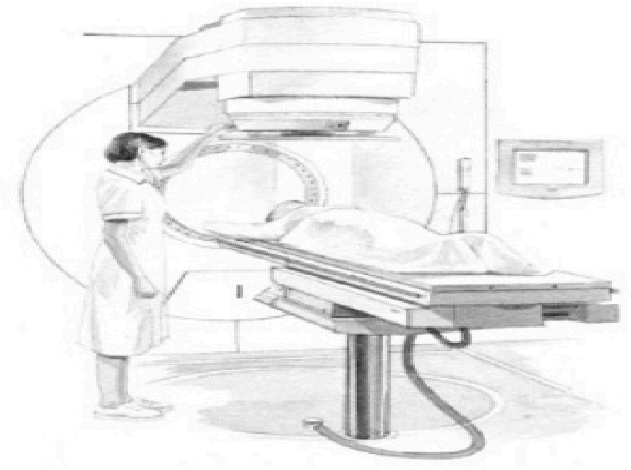

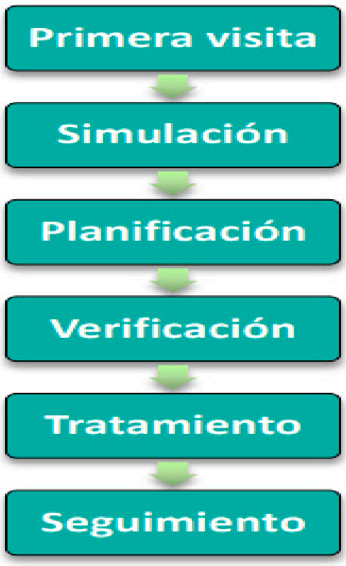

Supplement: Supplementary file 1 [file life-16-00029-s001.zip › life-4024333-supplementary.pdf]
